# A randomized, open-label, comparative efficacy trial of artemether-lumefantrine suspension versus artemether-lumefantrine tablets for treatment of uncomplicated *Plasmodium falciparum *malaria in children in western Kenya

**DOI:** 10.1186/1475-2875-7-262

**Published:** 2008-12-22

**Authors:** Elizabeth A Juma, Charles O Obonyo, Willis S Akhwale, Bernhards R Ogutu

**Affiliations:** 1 Kenya Medical Research Institute, Centre for Global Health Research, P. O. Box 1578 – 40100, Kisumu, Kenya; 2 Division of Malaria Control, Kenya Ministry of Health, P. O. Box 20750 – 00200 KNH, Nairobi, Kenya; 3 Kenya Medical Research Institute, Centre for Clinical Research, P. O. Box 54840 – 00200 Nairobi, Kenya

## Abstract

**Background:**

Artemether/lumefantrine (AL) has been adopted as the treatment of choice for uncomplicated malaria in Kenya and other countries in the region. Six-dose artemether/lumefantrine tablets are highly effective and safe for the treatment of infants and children weighing between five and 25 kg with uncomplicated *Plasmodium falciparum *malaria. However, oral paediatric formulations are urgently needed, as the tablets are difficult to administer to young children, who cannot swallow whole tablets or tolerate the bitter taste of the crushed tablets.

**Methods:**

A randomized, controlled, open-label trial was conducted comparing day 28 PCR corrected cure-rates in 245 children aged 6–59 months, treated over three days with either six-dose of artemether/lumefantrine tablets (Coartem^®^) or three-dose of artemether/lumefantrine suspension (Co-artesiane^®^) for uncomplicated falciparum malaria in western Kenya. The children were followed-up with clinical, parasitological and haematological evaluations over 28 days.

**Results:**

Ninety three percent (124/133) and 90% (121/134) children in the AL tablets and AL suspension arms respectively completed followed up. A per protocol analysis revealed a PCR-corrected parasitological cure rate of 96.0% at Day 28 in the AL tablets group and 93.4% in the AL suspension group, p = 0.40. Both drugs effectively cleared gametocytes and were well tolerated, with no difference in the overall incidence of adverse events.

**Conclusion:**

The once daily three-dose of artemether-lumefantrine suspension (Co-artesiane^®^) was not superior to six-dose artemether-lumefantrine tablets (Coartem^®^) for the treatment of uncomplicated malaria in children below five years of age in western Kenya.

**Trial registration:**

ClinicalTrials.gov NCT00529867

## Background

Resistance to the commonly used anti-malarial drugs has probably contributed substantially to the increased morbidity and mortality due to malaria [1]. As in many sub-Saharan countries, resistance to chloroquine (CQ) and subsequently resistance to sulphadoxine-pyrimethamine (SP) constrained malaria control efforts in Kenya. In 2004, the Kenyan Ministry of Health promulgated a change in the first-line treatment of uncomplicated malaria, replacing SP with the fixed-dose combination of artemether-lumefantrine tablets (Coartem^®^), which, at the time, was the only co-formulated artemisinin-based combination therapy (ACT) [2].

Previous studies in Africa have shown that the six-dose regimen of artemether-lumefantrine (AL) tablets is safe, efficacious and effective in the treatment of uncomplicated malaria in children less than five years of age, residing in areas with high levels of CQ and SP resistance [3-8]. Subsequently, several countries in sub-Saharan Africa have adopted AL as the treatment of choice for uncomplicated malaria. However, the implementation of this treatment policy change has faced several programmatic challenges in Kenya and Zambia. There was at least a two-year time lag between policy change and early implementation, partly due to lack of sustainable financing and weak health systems. Initial studies have cited lower degrees of preparedness by health facilities and workers resulting in sub-optimal quality of treatment at the point of care, due to irregular drug supply, inconsistent training and lack of supervision [2,9-11].

Although children less than five years of age are the major target of anti-malarial drug therapy in malaria endemic regions, the available oral paediatric formulations of ACT are not optimal for this high-risk population; young children cannot swallow whole tablets and sometimes spit out the drug, because of the bitter taste of the crushed tablets. In addition, AL tablets are not recommended for patients weighing less than 5 kg. These drug administration problems influence prescription of ACT by health workers and patient adherence, resulting in either under-dosing or over-dosing. Within the context of home-based management of malaria, 10 – 20% of participants were either non-adherent or administered incorrect doses of AL tablets [12-14]. There is an urgent need for oral preparations of ACTs for young children, which are easy to administer and stable under tropical conditions. Evaluation of an AL paediatric dispersible tablet has been completed, showing good efficacy (Salim Abdulla, personal communication). Artemether/lumefantrine powder for suspension is a fixed-dose combination of the two anti-malarials developed by Dafra Pharma NV in 2004 and manufactured by Manufacturing Packaging Farmaca (MPF) b.v. in the Netherlands under GMP. AL suspensionwas shown to be safe and efficacious in pre-registration trials in paediatric patients in Zambia, Sudan and Ivory Coast [15-17] (Additional file 1).

## Materials and methods

### Study site

This study was conducted at Chulaimbo Health Centre in Kisumu District in western Kenya. The facility serves a predominantly rural population in an area with high perennial malaria transmission in the lowlands around Lake Victoria. Transmission peaks during the long rains (March–May) and the short rains (October–December). The annual entomological inoculation rate in the surrounding area was 31.1 infectious bites/person/year for 12 months ending June 2004 [18]. More than 95% of infections are caused by *P. falciparum*, with the remaining being mixed *P. falciparum *with *Plasmodium malariae *and rarely *Plasmodium ovale *infections. *In vivo *drug resistance to SP in children <5 years is 74.5% [19,20] and 59% to amodiaquine [21].

### Patient screening and recruitment

A 2003 WHO protocol for the assessment and monitoring of anti-malarial drug efficacy for the treatment of uncomplicated falciparum malaria was used [22]. Children presenting to the health facility were screened for eligibility and invited to participate in the study if they met the following inclusion criteria: aged 6–59 months; body weight ≥ 5 kg; a history of fever in the previous 24 hours or measured fever (axillary temperature ≥ 37.5°C); monoinfection with *P. falciparum*, with parasitaemia in the range of 2,000–200,000 asexual parasites per microlitre of blood; no other cause for fever than suspected malaria; and, no general danger signs or signs of severe and complicated falciparum malaria as per WHO guidelines [23].

### Study design, randomization and treatment

A randomized, controlled, open-label trial design was used. PCR-corrected cure rate by day 28 after first dose was the primary endpoint used for computing sample size. Assuming a 94% cure rate with artemether-lumefantrine tablets and 99.9% with the artemether-lumefantrine suspension, a sample of 134 children was required (i.e. 127, including 5% adjustment for loss to follow up) in each treatment arm to detect this 6% difference in the parasitological cure rates, with 80% power using a two-sided alpha of 0.05. The randomization code was computer-generated without stratification from which treatment groups were assigned.

At enrolment, a medical history was obtained from parents/guardians including presenting symptoms, current medications, previous anti-malarial use and bed net use. A physical examination was performed, weight and axillary temperatures recorded and finger prick blood obtained for malaria smears, haemoglobin and blotted on filter paper for parasite genotyping.

Consecutively eligible children were randomly assigned into one of two treatment groups according to the randomization code. A study nurse prepared and administered the study medications, according to the treatment assignment. One group received 6 doses of artemether/lumefantrine 20/120 mg tablets (Coartem^®^, Novartis AG), crushed, mixed with water and administered at hours 0, 8, 24, 36, 48 and 60 over 3 days while the second group received artemether/lumefantrine powder for suspension (Co-artesiane^® ^Dafra Pharma NV) containing 15 mg artemether and 90 mg lumefantrine per 5 ml after reconstitution. A bottle of suspension was prepared for each child and administered once daily at hour 0, 24 and 48 over 3 days. Treatment doses were calculated based on patient weight and the administration directly observed under in-patient care. All children were given a glass of milk or breast fed (for those still breastfeeding) after drug administration since treatment times did not always coincide with meals. They were then observed for 30 minutes after drug administration for vomiting. If vomiting occurred, the whole treatment dose was re-administered. If the re-treatment dose was vomited, rescue treatment with parenteral quinine was administered and the child was withdrawn from the study.

All treatment failures were treated with oral or parenteral quinine for 7 days depending on clinical presentation. Paracetamol was administered to children with temperature ≥ 38.0°C or at the clinician's discretion. Antihelminthics were given to all children >1 year who had not received any in the past 3 months while children with haemoglobin <10 g/dl were treated with ferrous sulphate for 14 days. Long lasting insecticide treated bed nets (Olyset^®^, Sumitomo Corp) were given to the children on completion of the study.

### Follow-up

All children in the study were admitted to hospital for 3 days for observed drug administration and followed up for a total of 28 days. Clinical and parasitological evaluation was performed during hospitalization (days 0, 1, 2 and 3) and at the study clinic during scheduled visits on days 7, 14, and 28, after initiating study treatment, or on any other day if the child was unwell. During each scheduled visit, a brief clinical history was obtained and a physical examination was performed; blood smears for malaria parasites and filter paper spot samples were obtained. Patients who did not return to the clinic for scheduled visits by mid day were visited at home by the social worker and asked to come to the health facility. Patients were excluded from the study if they; 1) withdrew consent, 2) left the study area, or 3) reported taking anti-malarial medication during follow-up.

### Laboratory evaluation

Blood was drawn from a finger prick to prepare thick and thin blood smears on days 0, 1, 2, 3, 7, 14, 28 and on any other unscheduled visit. The smears were air dried, stained with 3% Giemsa for 30 – 45 minutes and read independently by two technologists. Parasite density was calculated by counting asexual parasites against a 500 leucocytes assuming a leukocyte count of 8,000/μL of blood to obtain number of parasites and gametocytes per microlitre (μL). Thin smears were examined for plasmodium parasite speciation. A slide was considered negative after scanning 100 high power fields. A third microscopist independently read discrepant slides. Haemoglobin was measured using HemoCue^® ^(HemoCue AB Angelholm, Sweden) on Days 0, 7, 14 and 28. Filter paper blood spots were collected on Days 0, 14 and 28 or any other day of recurrent parasitaemia. Paired filter paper samples of children who had parasitaemia during follow-up were used to extract parasite DNA for PCR to distinguish recrudescent from new infections as described by Snounou et al. [24]. Block 2 of MSP-1 and block 3 of MSP-2 were amplified by nested PCR and size polymorphisms identified by gel electrophoresis against a 100-basepair (bp) molecular weight marker (New England Biolabs, Beverly, MA). Parasites were classified as recrudescent if they shared any of the bands that were present on day 0 and as new infections if there were no common bands.

### Ethical approval

This study was approved by the Kenya National/Kenya Medical Research Institute Ethics Committee. Written informed consent was obtained from all parents or guardians of eligible children prior to enrolment.

### Statistical analysis

Data from case report forms were checked, double entered and verified for errors using Epi Info 2002 (Centers for Disease Control and Prevention, Atlanta, GA USA). Data was analysed using SPSS (v.12, SPSS Inc. Chicago, IL USA) and Epi-Info 2002. The primary efficacy endpoint was day 28 PCR-corrected parasitological cure rate, defined as the proportion of patients without asexual parasitaemia within 7 days after beginning treatment, without recrudescence within 28 days after beginning treatment and who demonstrated no need for rescue treatment for signs of clinical malaria within 28 days after initiation of study treatment. Secondary end-points included PCR-corrected parasitological cure rate on day 14, gametocyte carriage, fever and parasite clearance rate. The intent to treat (ITT) population defined as all randomized patients who took at least one dose of study medication was used for safety analysis. Proportions were compared between treatment groups using the chi-square test. Normally distributed variables were compared using the Student's *t*-test and analysis of variance (ANOVA). For skewed data, medians were computed and comparisons made using the Kruskal-Wallis one way ANOVA. Two tailed p-values < 0.05 were considered statistically significant.

## Results

### Participant characteristics

**Figure 1:**
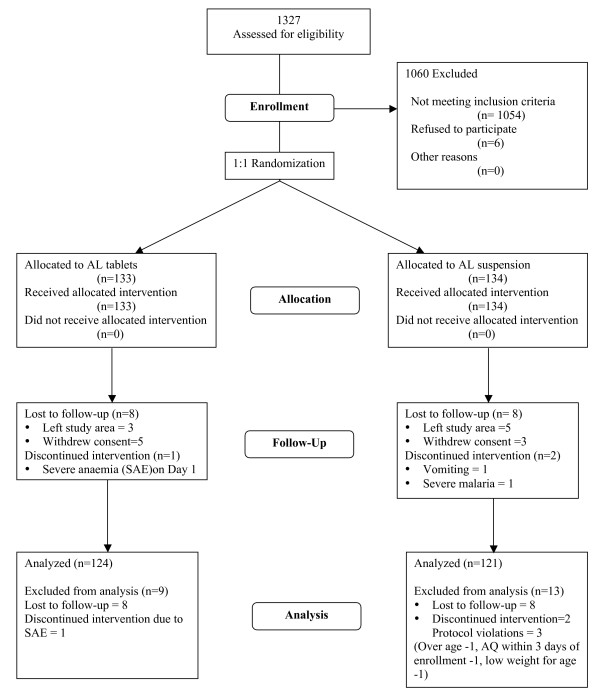
**The trial profile**.

Two hundred and sixty seven of the 1327 children screened for malaria were enrolled into the trial; 134 in the AL suspension arm and 133 in the AL tablets arm. Seventeen (6.4%) children withdrew or were lost to follow up. Three (1.1%) children, all from the AL suspension arm, were excluded from analysis due to age >59 months, low weight for age and treatment with amodiaquine within three days of enrolment. Two children in the AL suspension arm were withdrawn from treatment due to repeated vomiting on Day 0. Data from 245 children was analysed; 124 in the AL tablets arm and 121 in the AL suspension arm (Figure 1). Both treatment groups were comparable in terms of baseline demographic, clinical and laboratory characteristics (Table 1).

**Table 1:** Baseline characteristics of children enrolled in the trial

Characteristic	AL tablets N = 124	AL suspension N = 121	p
Female sex (%)	65 (52.4)	58 (47.9)	0.52

Prior anti-malarial use (2 months) N (%)	3 (2.4)	1 (0.8)	0.62

ITN use N (%)	55 (44.4)	58 (47.9)	0.49

Median age in months (+ SD)	28 (16.3)	25 (15.6)	0.63

Mean weight in kg (+ SD)	11.4 (3.3)	11.7 (3.1)	0.38

Mean temperature °C (+ SD)	38.1 (1.2)	38.2 (1.2)	0.70

Mean* parasitaemia per μl (range)	25,231 (2008 – 195288)	34,881 (2111 – 196343)	0.37

Mean haemoglobin g/dl (+ SD)	9.5 (2.1)	9.7 (1.9)	0.38

Proportion with gametocytes N (%)	14 (11.2)	10 (8.2)	0.42

Mean dose A/L mg (SD)	124 (49)/871 (294)	121 (40)/807 (239)	0.06

### Parasitological cure rates

There were two (0.7%) early treatment failures. One child in the AL tablets arm developed severe anaemia (Hb < 5.0 g/dl) on Day 1 and was withdrawn from treatment while another child in the AL suspension arm developed convulsions on Day 0 and received treatment for severe malaria. Both children recovered after treatment. The PCR corrected cure rate for day 14 was 100% in the AL tablets arm and 98.4% in the AL suspension arm while the day 28 cure rates were 96% (95% 95% CI 90.8 – 98.7) in the AL tablets arm and 93% (95% CI 87.4 – 97.1) in the AL suspension arm p = 0.40 (Table 2). The re-infection rate was 9.6% in the AL tablets arm and 5.7% in the AL suspension arm (p = 0.25).

**Table 2:** PCR corrected clinical and parasitological responses on Days 14 and 28

	AL tablets	AL suspension	p
Day 14 cure rate, no. (%)	127/127 (100)	122/124 (98.4)	0.24

Day 28 cure rate, no. (%)	119/124 (96.0) 95% CI 90.8 – 98.7	113/121 (93.4) 95% CI 87.4 – 97.1	0.40

### Fever, parasite and gametocyte clearance

**Figure 2:**
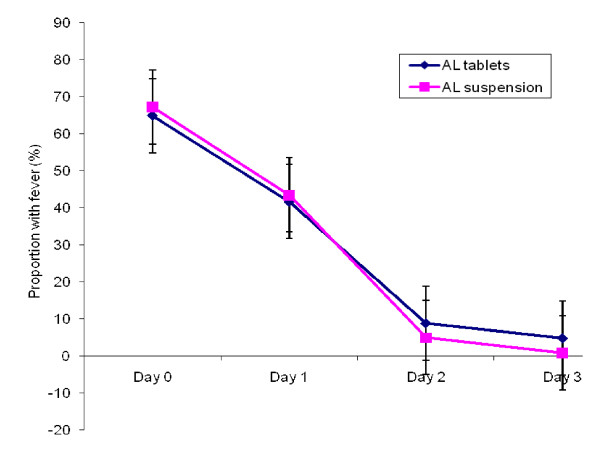
**Proportion of children with fever during treatment**.

**Figure 3:**
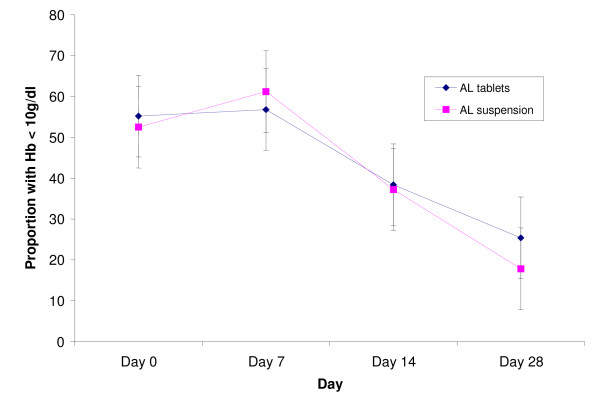
**Proportion of children with haemoglobin <10 g/dL**.

**Figure 4:**
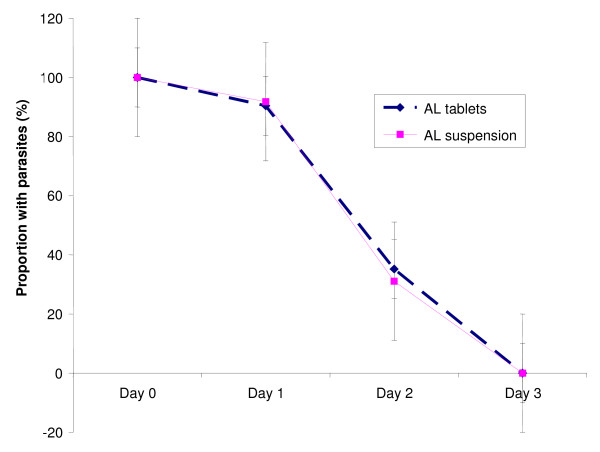
**Proportion of children with parasites during the first three days after treatment**.

The proportions of children with fever (temperature ≥ 37.5°C), and parasitaemia over the first four days, and anaemia (haemoglobin < 10 g/dl) over the 28 day follow-up period were similar in both treatment groups (Figures 2, 3, 4). Fourteen (11.2%) and 10 (8.2%) of children in the AL tablets and AL suspension arms, respectively, had gametocytes on Day 0. Both treatments were effective in clearing gametocytes with only one participant in each treatment arm having gametocytes on Day 7 and none by Day 28.

### Adverse events

There was no difference in the incidence of vomiting between the treatment groups as a proportion of treatment doses administered over the first 3 days of treatment: AL tablets arm, 21/807 (2.6%) and AL suspension arm, 12/406 (3.0%), p = 0.72. Two patients (one in each arm) had diarrhoea, which was suspected to be due to the drugs.

Seven Serious Adverse Events (SAE) were recorded during the trial, four in the AL tablets arm and three in the AL suspension arm, none of which was considered related to the study drugs. Three (2.4%) children in the AL tablets arm developed severe anaemia (haemoglobin<5.0 g/dl) during the study. One child developed severe anaemia on Day 1 and was withdrawn from the intervention, while the other two had severe anaemia detected on Day 7 during a routine follow-up visit. All three children with severe anaemia were enrolled with haemoglobin between 5–7 g/dl and were treated with iron supplementation only. The fourth child (0.8%) developed severe malaria on day 26 during follow-up. In the AL suspension arm, one child developed severe malaria and meningitis on Day 0 and was withdrawn from treatment, he subsequently developed neurological sequelae. The other children each developed severe malaria (day 22) and severe pneumonia (day 28) and recovered completely. Other adverse events are summarized in Table 3.

**Table 3:** Summary of Adverse Events

Adverse event	AL tablets n = 133* (%)	AL suspension n = 134* (%)	*p*
Diarrhoea	11 (8.3)	10 (7.4)	0.81

Vomiting during treatment**	21 (2.6)	12 (3.0)	0.72

Rhinitis	17(12.8)	22 (16.4)	0.40

Otitis Media	1 (0.8)	5 (3.7)	0.21

Pneumonia	3 (2.3)	6 (4.5)	0.50

Bacterial skin infections	5 (3.8)	5 (3.7)	1.00

Fungal skin infections	6 (4.5)	5 (3.7)	0.98

Eczematous rash	1(0.8)	5 (3.7)	0.21

Contact dermatitis	1 (0.8)	1 (0.7)	1.00

Conjunctivitis	6 (4.5)	7 (5.2)	0.98

* n = total number of children randomized to the treatments

** Vomiting episodes as a proportion of number of treatment doses administered

## Discussion

The 28 day PCR corrected efficacy of AL suspension was 93.4% compared with 96.0% for AL tablets (p = 0.40). Previous trials reported efficacy rates between 98 – 100% for AL suspension in various parts of Africa [15-17]. The efficacy of the AL tablets in this study was consistent with 95.4% efficacy obtained in the same region in 2004 prior to introduction of AL as first line treatment for uncomplicated malaria in Kenya (Juma et al, unpublished data).

The process of changing national treatment policies for malaria is complex and the costs involved are substantial [2,25,26]. Conceivably, it may be equally difficult for both health workers and caretakers of sick children to implement a change in drug formulations. Proper conduct of such policy changes should be evidence-based. This study provides some evidence for policy and clinical practice in use of AL as the first line treatment in Kenya and like countries in the region. For the first time, an investigation was carried out to compare the efficacy and safety of the AL suspension with the AL tablets, and no evidence was found that the suspension is less efficacious. Both AL preparations have a fixed-dose artemether to lumefantrine ratio of 1:6 with doses calculated to give an average of 4 mg/kg artemether per day. The AL tablet requires twice daily doses while the AL suspension has the advantage of single daily administration. A previous study also found that the AL suspension is safe to administer to children below 5 kg of weight [15]. One disadvantage of the suspension is the narrow weight range (2.0 kg for given dose – which calls for proper reconstitution and good dispensing practice) compared with 10 kg for a given dose of AL tablets.

The major motivation to evaluate this suspension was the limitations of administering hard tablets to children especially the very young, by mothers and health workers. These difficulties notwithstanding, AL tablets are efficacious and effective in this age-group. Although not widely available, an AL dispersible tablet for paediatric patients is currently under review by regulatory authorities. AL suspension and probably the AL paediatric dispersible tablet will be useful alternatives and a milestone in the treatment of uncomplicated malaria in young children who are unable to take AL tablets. Further studies are needed to evaluate the effect of widening the weight-based dosing ranges of AL suspension for ease of dispensing and administration. This study did not focus on the safety by body weight and future studies should investigate this aspect (see additional file 2).

PCR-corrected cure rates were used as the primary efficacy endpoint. Coupled with a comparative randomized design, the results obtained in the trial reflect a valid measure of the AL suspension's efficacy and also provides evidence of non-declining efficacy of AL in the treatment of uncomplicated malaria in children in western Kenya known to be the hot bed of anti-malarial drug resistance in Africa. There were two recrudescent parasitological treatment failures in children on the AL suspension arm at Day 14. These children did not vomit or spit out the study medication, had levels of parasitaemia of 4376/μl and 42,640/μl respectively and were free of parasites by day-2 of treatment. All previous studies have reported 100% ACPR at day-14 with the AL suspension. The reasons for this are unclear and may need to be investigated further, especially day-7 lumefantrine blood levels. AL suspension is given once daily and lumefantrine absorption can be erratic in some children [27]. As expected both treatment regimens were effective in shortening the fever, parasite clearance times and reduction of gametocyte carriage.

Both regimens were well tolerated with the incidence of vomiting in the first three days of treatment being similar for both groups. Other adverse events reported during follow-up occurred similarly between treatment groups and included respiratory infections (21.7%), malaria (11.2%) and skin infections (8.6%), none attributed to the study medications.

This trial had several limitations. The open label design could bias the results because the parents of the children were aware of their children's treatment assignment. Children were not followed up to 42 days as recommended by WHO guidelines [22]. However, the population in the catchment area of our study site is exposed to high perennial transmission of malaria and a longer follow-up period would most likely show more cases with re-infection than recrudescence to impact final efficacy outcomes. The power of this study computed post hoc was only 65%, indicating that the sample size studied was not sufficient to detect a significant difference in efficacy between the two treatment formulations. Furthermore, this study was conducted under supervised conditions and may not reflect the realities of treatment practices in normal outpatient care. Larger trials to confirm non-inferiority between these two formulations are recommended.

## Conclusion

The once daily 3-dose of artemether-lumefantrine suspension (Co-artesiane^®^) was not superior to 6-dose artemether-lumefantrine tablets (Coartem^®^) for the treatment of uncomplicated malaria in children below 5 years of age in western Kenya.

## Abbreviations

ACPR: Adequate Clinical and Parasitological Response; ACT: Artemisinin based Combination Therapies; AL: Artemether/Lumefantrine; CDC: Centres for Disease Control and Prevention; ETF: Early Treatment Failure; KEMRI: Kenya Medical Research Institute; LCF: Late Clinical Failure; LPF: Late Parasitological Failure; LTF: Late Treatment Failure; PCR: Polymerase Chain Reaction; SP: Sulphadoxine/Pyrimethamine; WHO: World Health Organization

## Competing interests

Elizabeth Juma and Charles Obonyo received payments to attend meetings related to the trial.

## Authors' contributions

EJ was responsible for proposal development, study design, training of research assistants, data analysis and development and submission of this manuscript. CO, BO and WA participated in protocol development, training, and field supervision of the trial and revision of the manuscript for publication.

## Supplementary Material

Additional file 1 Short report by the sponsor Dafra Pharma nv/sa. A relative bioavailability study for Fixed Dose Combination (FDC) comparing Coartem Tablets (containing Artemether 20 mg and Lumefantrine 120 mg) of Novartis Pharmaceuticals Limited, EU with Co-Artesiane^® ^dry powder for suspension (containing β-Artemether 360 mg and Lumefantrine 2160 mg in 45.6 g dry powder for suspension of 120 ml) of Dafra Pharma NV, Belgium in 42 + 6 healthy adult human subjects. Click here for file

Additional file 2 **Discussion Dosing Al**. Following the WHO guidelines for the treatment of malaria (WHO, 2006, pages 23–24) the recommended dose for artemether/lumefantrine tablets (Coartem^®^) when used for children between 5 and 14 kg is 1 tablet at time 0 h and 1 tablet at time 8 h followed by two tablets a day for two days (24, 36, 48 and 60 h). Calculated as mg artemether per kg body weight (bw), this means that for a child of 5 kg a dose of 8 mg artemether/kg per day spread over two doses is given. Evidence that this high dose of more than 4 mg/kg bw of artemether is needed for small babies (± 5 kg) is scanty or non existent. Moreover, this "overdosing" situation is in contrast with the adult dose of 8 pills a day divided over two intakes. This means that an adult of 50 kg takes a dose of 3.2 mg/kg of artemether and an adult of 75 kg takes one of 2.1 mg/kg per day. There is no dose finding study published that we could find to justify this variation in dosing. Click here for file
