# Supplementary material for: A randomized, open-label, comparative efficacy trial of artemether-lumefantrine suspension versus artemether-lumefantrine tablets for treatment of uncomplicated Plasmodium falciparum malaria in children in western Kenya
Source: Malar J. 2008 Dec 22;7:262. doi: 10.1186/1475-2875-7-262 (PMC2635380; doi:10.1186/1475-2875-7-262)
Supplement: Additional file 1 — Short report by the sponsor Dafra Pharma nv/sa. A relative bioavailability study for Fixed Dose Combination (FDC) comparing Coartem Tablets (containing Artemether 20 mg and Lumefantrine 120 mg) of Novartis Pharmaceuticals Limited, EU with Co-Artesiane® dry powder for suspension (containing β-Artemether 360 mg and Lumefantrine 2160 mg in 45.6 g dry powder for suspension of 120 ml) of Dafra Pharma NV, Belgium in 42 + 6 healthy adult human subjects. [file 1475-2875-7-262-S1.pdf]

A relative bioavailability study for Fixed Dose Combination (FDC) comparing Coartem Tablets (containing Artemether 20 mg and Lumefantrine 120 mg) of Novartis Pharmaceuticals Limited, EU with Co-Artesiane® dry powder for suspension (containing β-Artemether 360 mg and Lumefantrine 2160 mg in 45.6 g dry powder for suspension of 120 ml) of Dafra Pharma NV, Belgium in 42 + 6 healthy adult human subjects

Short report by the sponsor Dafra Pharma nv/sa
Date: 14/10/2008

BASED ON THE OFFICIAL REPORT OF
BOMBAY BIO-RESEARCH CENTRE
DATE: 10th March 2008

Investigators:

Director & Quality Assurance Head:
Dharmendra Ved, M.Sc

Associate Director & Analytical Investigator:
Shrikant Rane, M.Sc. Ph.D

Principal Investigator: Prashant Kulkarni M.D

Study centre:
Clinical, Analytical and Statistical Facility

Bombay Bio-Research Centre
Plot No. 35, Deonar Ancillary Industrial Plots,
Govandi, Mumbai-400 043, India

Pathological Testing Facility:
Clinical Diagnostic Centre
F 11 & F 12/1, Ground Floor,
Opp. SEEPZ Main gate,
Andheri (East), Mumbai -400 093, India

**Study period:**	Enrolment date
**Date of First enrolment:**	03rd February 2007
**Date of Last completed sample:**	06th March 2007
**Phase of Development:**	Relative Bioavailability

OBJECTIVE

The study was designed to compare the rate and extent of absorption and safety of Coartem® tablets (containing Artemether 20 mg and Lumefantrine 120 mg) of Novartis Pharmaceuticals Limited, EU with Co-artesiane® dry powder for suspension (containing β-Artemether 360 mg and Lumefantrine 2160 mg in 45.6 g dry powder for suspension of 120 ml) of Dafra Pharma NV, Belgium in 42 + 6 healthy adult human subjects.

METHODS

Number of Subjects Recruited and Completed:

Total 42 (+ 6) healthy adult human subjects who met all inclusion and none of the exclusion criteria were recruited in the trial. 46 subjects completed the study. During period I, one subject was dropped from the study before dosing, due to violation of laboratory rules and a second subject did not report to the centre during check-in of period I. Samples for all the 46 subjects were analysed and data of 42 subjects is reported.

Diagnosis and main criteria for inclusion:

Diagnosis: The clinical safety was evaluated by recording the ECG, vital signs and adverse events. The laboratory safety was evaluated by recording routine laboratory tests including biochemistry, haematology and urine analysis before and after the study for all recruited subjects.

Main criteria for inclusion: Healthy adult human subjects within 18-45 years of age (inclusive).

Test Product, dose and mode of administration, batch number:

Test Product: Co-artesiane® (Artemether and Lumefantrine) dry powder for suspension

Dose: 53 ml of the suspension to obtain a dose of 160 mg Artemether and 960 mg Lumefantrine

Mode of Administration: Oral (administered with 240 mL of water)

Batch No.: 15099

Reference Product, dose and mode of administration, batch number:

Reference Product: Coartem® (Artemether and Lumefantrine) tablets **Dose:** 8 Tablets to obtain a dose of 160 mg Artemether and 960 mg Lumefantrine **Mode of Administration:** Oral (administered with 240 mL of water) **Batch No.:** X0372

Duration of treatment:
A gap of 21 days was maintained between Period I and Period II dosing.

Analytical Methodology:

Artemether and its metabolite and Lumefantrine from plasma were quantified using a validated LC-MS/MS method.

Criteria for evaluation:

Primary Pharmacokinetic Parameters: C_{max}, AUC_{0-216} and AUC_{0-inf}, were evaluated for % Ratio and 90% CI from Log transformed data. **Secondary Pharmacokinetic Parameters:** T_{max}, k_{el} and $t_{1/2}$ were evaluated for % Ratio.

Statistical Method:
ANOVA, two one-sided tests for bioequivalence, 90% CI and ratio analysis for untransformed and log-transformed pharmacokinetic parameters C_{max}, AUC_{0-216} and AUC_{0-inf} were performed.

Dafra Pharma nv/sa
Slachthuisstraat 30 bus 7
2300 Turnhout
Belgium

Tel: ++32-(0)14-61.78.20
Fax: ++32-(0)14-61.78.20
E-mail: info@dafra.be
website: www.dafra.be

SUMMARY - CONCLUSIONS

Primary Pharmacokinetic Parameters:

ARTEMETHER

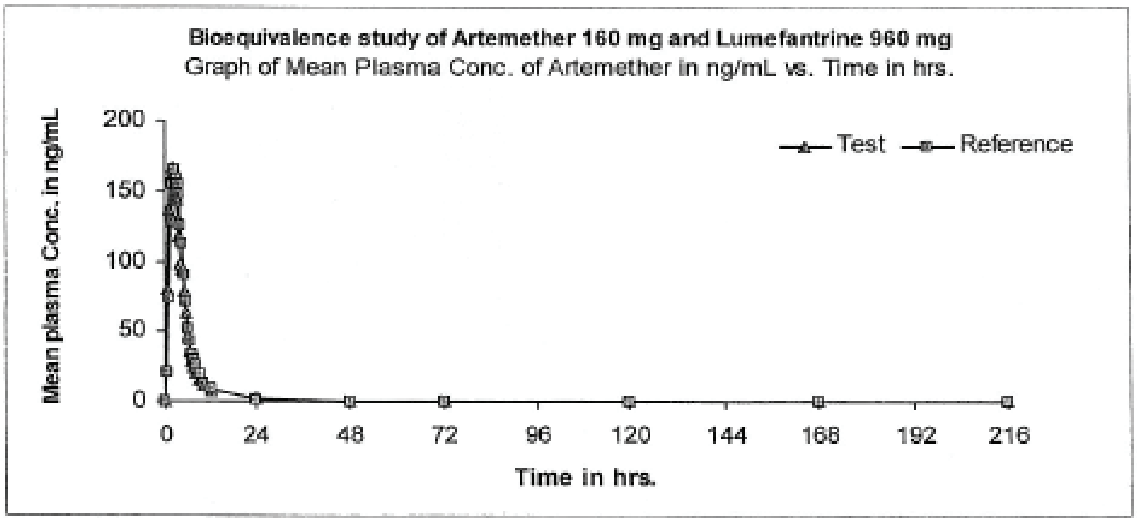

% Ratio of Untransformed Data

Primary Parameter	Test Product	Reference Product	% Ratio of Test to Reference Product
C_{max} (ng/mL)	197.96	201.73	98.13
AUC_{0-216} (ng × hr/mL)	846.252	851.140	99.43
AUC_{0-inf} (ng × hr/mL)	855.577	860.945	99.38

90 % Confidence Interval from Log Transformed Data

Pharmacokinetic Parameters	Acceptance Criteria	% Confidence Interval
LnC_{max}	80-125%	92.57 - 107.00
$LnAUC_{0-216}$	80-125%	90.87 - 110.04
$LnAUC_{0-inf}$	80-125%	90.98 - 109.78

CONCLUSION: Based on the above data, the Test Product meets the criteria for bioequivalence (primary pharmacokinetic parameters were within acceptance criteria of 80 to 125%), when compared with Reference Product with respect to Artemether.

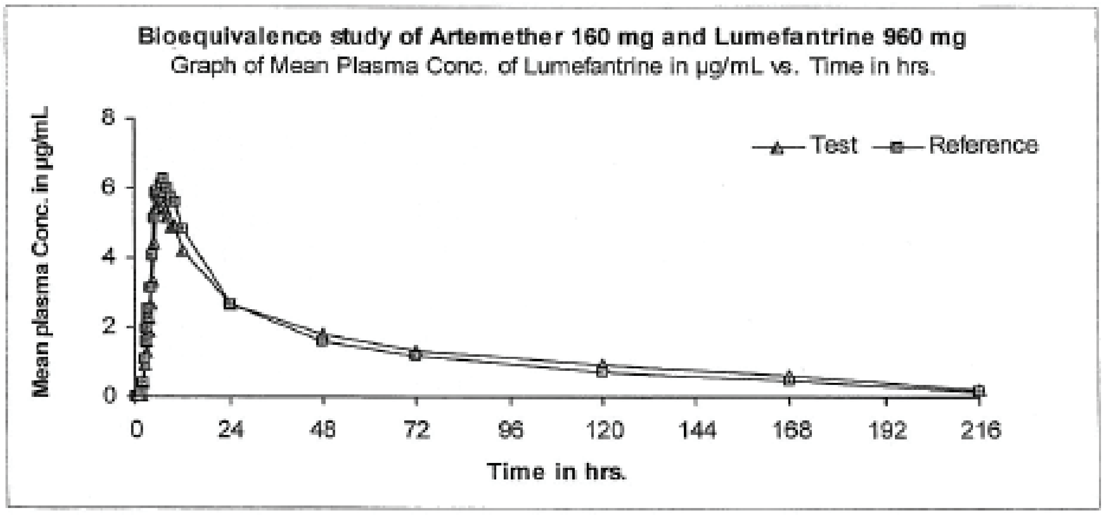

% Ratio of Untransformed Data

Primary Parameter	Test Product	Reference Product	% Ratio of Test to Reference Product
C_{max} (µg/mL)	7.50	6.96	107.84
AUC_{0-216} (µg × hr/mL)	283.987	263.580	107.74
AUC_{0-Inf} (µg × hr/mL)	285.810	266.236	107.35

90 % Confidence Interval from Log Transformed Data

Pharmacokinetic Parameters	Acceptance Criteria	% Confidence Interval
LnC_{max}	80-125%	96.46 -119.47
$LnAUC_{0-216}$	80-125%	95.54 - 118.28
$LnAUC_{0-inf}$	80-125%	95.17 - 117.32

CONCLUSION: Based on the above data, the Test Product meets the criteria for bioequivalence (primary pharmacokinetic parameters were within acceptance criteria of 80 to 125%), when compared with reference product with respect to Lumefantrine.

Secondary Pharmacokinetic Parameters:

ARTEMETHER

Secondary Parameter	Test Product	Reference Product
T_{max} (hrs.)	2.05	2.67
k_{el}	0.544	0.528
$t_{1/2}$	1.87	2.34

LUMEFANTRINE

Secondary Parameter	Test Product	Reference Product
T_{max} (hrs.)	7.15	6.83
k_{el}	0.057	0.040
$t_{1/2}$	37.65	40.65

Safety Parameters:

No major abnormalities or variations were reported due to test or reference medication in subject clinical and laboratory parameters. The overall conclusion was that both the medications at said dose are safe for administration.
