# Supplementary material for: A randomized, open-label, comparative efficacy trial of artemether-lumefantrine suspension versus artemether-lumefantrine tablets for treatment of uncomplicated Plasmodium falciparum malaria in children in western Kenya
Source: Malar J. 2008 Dec 22;7:262. doi: 10.1186/1475-2875-7-262 (PMC2635380; doi:10.1186/1475-2875-7-262)
Supplement: Additional file 2 — Discussion Dosing Al. Following the WHO guidelines for the treatment of malaria (WHO, 2006, pages 23–24) the recommended dose for artemether/lumefantrine tablets (Coartem®) when used for children between 5 and 14 kg is 1 tablet at time 0 h and 1 tablet at time 8 h followed by two tablets a day for two days (24, 36, 48 and 60 h). Calculated as mg artemether per kg body weight (bw), this means that for a child of 5 kg a dose of 8 mg artemether/kg per day spread over two doses is given. Evidence that this high dose of more than 4 mg/kg bw of artemether is needed for small babies (± 5 kg) is scanty or non existent. Moreover, this "overdosing" situation is in contrast with the adult dose of 8 pills a day divided over two intakes. This means that an adult of 50 kg takes a dose of 3.2 mg/kg of artemether and an adult of 75 kg takes one of 2.1 mg/kg per day. There is no dose finding study published that we could find to justify this variation in dosing. [file 1475-2875-7-262-S2.pdf]

DISCUSSION DOSING AL

28/08/2007

Following the WHO guidelines for the treatment of malaria (WHO, 2006, pages 23-24) the recommended dose for artemether/lumefantrine tablets (Coartem®) when used for children between 5 and 14 kg is 1 tablet at time 0h and 1 tablet at time 8h followed by two tablets a day for two days (24, 36, 48 and 60h). Calculated as mg artemether per kg body weight (bw), this means that for a child of 5 kg a dose of 8 mg artemether/kg per day spread over two doses is given. Evidence that this high dose of more than 4 mg/kg bw of artemether is needed for small babies (± 5kg) is scanty or non existent. Moreover, this "overdosing" situation is in contrast with the adult dose of 8 pills a day divided over two intakes. This means that an adult of 50 kg takes a dose of 3.2 mg/kg of artemether and an adult of 75 kg takes one of 2.1 mg/kg per day.

There is no dose finding study published that we could find to justify this variation in dosing.

WHO recommendation (WHO, 2006, page 24):

Table 1. Dosing schedule for artemether-lumefantrine

Body weight in kg (age in years)	No. of tablets at approximate timing of dosing[a]					
	0 h	8 h	24 h	36 h	48 h	60 h
5–14 (<3)	1	1	1	1	1	1
15–24 (≥3–8)	2	2	2	2	2	2
25–34 (≥9–14)	3	3	3	3	3	3
>34 (>14)	4	4	4	4	4	4

[a] The regimen can be expressed more simply for ease of use at the programme level as follows: the second dose on the first day should be given any time between 8 h and 12 h after the first dose. Dosage on the second and third days is twice a day (morning and evening).

We realize that we are at variance with the Novartis' recommended dose. We have based our Co-Artesiane® suspension dosage on a general dose of 4 mg artemether per kg bw, as recommended by the WHO for artesunate, and we give only one intake a day in stead of two.

1) General dose of 4 mg artemether per kg bw

As there is no evidence found in literature which would indicate the need to go higher or lower then the dose of 4 mg/kg bw a day, we tried to follow this acceptable recommendation. In the WHO guidelines (WHO, 2006, pages 24, 25) it is clearly stated for artesunate that the total recommended dose given per day for three days is 4 mg/kg bw. The pharmacokinetics of artesunate and artemether are quite similar (Newton *et al.*, 2000 and Silamut *et al.*, 2003). Artesunate and artemether can be considered as prodrugs for dihydroartemisinin. Biotransformation into the active metabolite dihydroartemisinin occurs rapidly, almost immediately for artesunate and somewhat later for artemether. The reported elimination half-life of artesunate is less than 1 hour (Newton *et al.*, 2000) and for artemether between ± 2 hours (Silamut *et al.*, 2003). The dose of artemether necessary to obtain antimalarial activity is considered to be the same for artesunate, which is 4 mg/kg bw per day for 3 days as stated in the WHO guidelines for Artesunate (WHO, 2006, pages 24, 25).

Dafra Pharma's syrup formulation allows to precisely follow the recommended dose of 4 mg/kg bw artemether. Dafra Pharma calculated for different body weights how many millilitres of the suspension should be administered to obtain a daily dose of 4 mg/kg Artemether. We recommend to round off the dosage to the nearest subdivision. A subunit of 5 ml of the 60 or 120 ml bottle contains 15 mg artemether and 90 mg lumefantrine.

Body Weight	Daily Dose (ml)	Artemether mg/kg bw	
5kg	7 ml	4,2	60 ml bottle
6kg	8 ml	4,0	
7kg - 8kg	10 ml	(4,3 - 3,8)	
9kg -10kg	13 ml	(4,3 - 3,9)	
11kg -12kg	15 ml	(4,1 - 3,8)	
13kg -14kg	18 ml	(4,1 - 3,9)	
15kg - 17kg	22 ml	(4,4 - 3,9)	120 ml bottle
18kg - 20kg	25 ml	(4,1 - 3,8)	
21kg - 23kg	29 ml	(4,1 - 3,8)	
24kg - 26kg	33 ml	(4,1 - 3,8)	
27kg - 29kg	37 ml	(4,1 - 3,8)	
30kg	40 ml	4,0	

Dosing can easily be done with the marked (in millilitres) dosing cups.

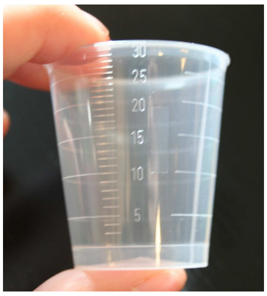

If we calculate for every kg bw how many mg artemether is given at the recommended dose of Coartem® and compare those values with those obtained with Co-Artesiane® , we see the following picture:

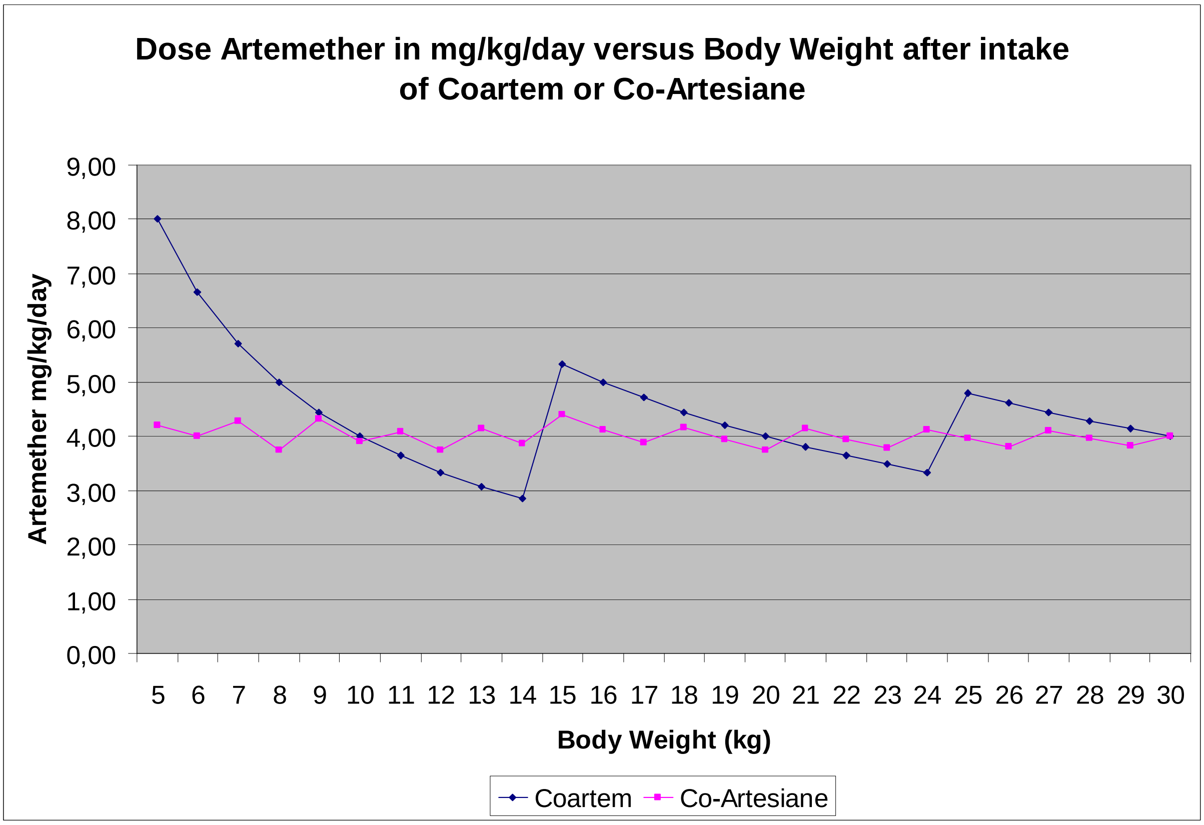

This graph demonstrates that, in particular for smaller children, the Novartis recommended dosing will lead to a potential overdosing whereas for some children, particularly in the range of 12 – 14 kg underdosing could occur. In this graph it is shown that with the recommended dose of Coartem® a big variation in the dose of artemether given per day is obtained, especially with the smaller children in the body weight category of 5 to 14 kg. In the WHO guidelines (WHO, 2006, page 36) it is stated that dosing of antimalarials is traditionally based on body weight for both adults and children/infants. However, most of the current antimalarial formulations do not allow a correct dosing for children/infants due to the fact that the tablets are optimised for adults. This is clearly evident for the Coartem® tablets in the target group with a bw of 5 to 14 kg.

For that reason, Dafra Pharma developed a paediatric artemether-lumefantrine suspension that allows correct dosing for that target group. The suspension enables an adequate daily dosing

of artemether over the whole body weight range (5 to 30 kg). The syrup therefore allows dosing around the baseline of 4mg/kg artemether, considered by WHO as the necessary dose. Excessive overdosing for very small children and potential underdosing in some weight groups are avoidable. For example:

- Co-Artesiane®: 5 kg child receives a dose of 4.2 mg/kg artemether per day (correct dose) ↔ Coartem®: 5 kg child receives a dose of *8 mg/kg* per day (*overdosing*)

- Co-Artesiane®: 14 kg child receives a dose of 4.3 mg/kg artemether per day (correct dose) ↔ Coartem®: 14 kg child receives a dose of *2.86 mg/kg* per day (*underdosing*)

**Body Weight (kg)**	**Artemether mg/kg per day COARTEM®**	**Artemether mg/kg per day CO-ARTESIANE®**
5	8,00	4,20
6	6,67	4,00
7	5,71	4,29
8	5,00	3,75
9	4,44	4,33
10	4,00	3,90
11	3,64	4,09
12	3,33	3,75
13	3,08	4,15
14	2,86	3,86
15	5,33	4,40
16	5,00	4,12
17	4,71	3,88
18	4,44	4,17
19	4,21	3,95
20	4,00	3,75
21	3,81	4,14
22	3,64	3,95
23	3,48	3,78
24	3,33	4,12
25	4,80	3,96
26	4,61	3,81
27	4,44	4,11
28	4,29	3,96
29	4,14	3,83
30	4,00	4,00

2) One dose versus two doses a day

It was demonstrated in a Global Fund for HIV/AIDS and Malaria (GFATM) sponsored trial that the proposed dosing for the artemether/lumefantrine syrup results in excellent parasite and fever clearance and the Adequate Clinical and Parasitological Response (ACPR) was found to be 100% (95% CI 96.0;100) (Chanda, 2006). We agree with the study of Ashley et al. (2007) that single dosing of the combination artemether/lumefantrine per day would be better, however their results indicated that artemether-lumefantrine efficacy is reduced by once-daily dosing. Our results did clearly demonstrate that we achieved the same efficacy with single dosing per day of the Co-Artesiane® syrup then obtained with the twice daily dosing of the Coartem® tablets. This could be due to two facts: either there were strain differences of the parasite between the two studies (Asian study versus African study) or the absorption of lumefantrine out of the syrup is much better then the absorption out of the tablet. We therefore recommend the syrup with a single dose daily.

Considering however that a high plasma peak concentration of artemether and dihydroartemisinin is in favour of avoiding the induction of tolerance, we consider our proposed dosing schedule and scheme superior based on the WHO recommendations and all statements. Moreover, it is clearly stated in the WHO guidelines that a constant baseline blood plasma concentration of a malaria chemotherapeutic selects for resistance. Therefore, it is better to give a peak blood plasma concentration in order to get biological activity and to lower the risk of resistance.

The following graphs were calculated based on a bioequivalence study. It is clearly shown that the the peak plasma concentrations of artemether and dihydroartemisinin are higher with 3 intakes of Co-Artesiane® suspension than with the 6 intakes of Coartem®. Coartem® dosing leads to a low and constant baseline plasma concentration which could facilitate the induction of tolerance.

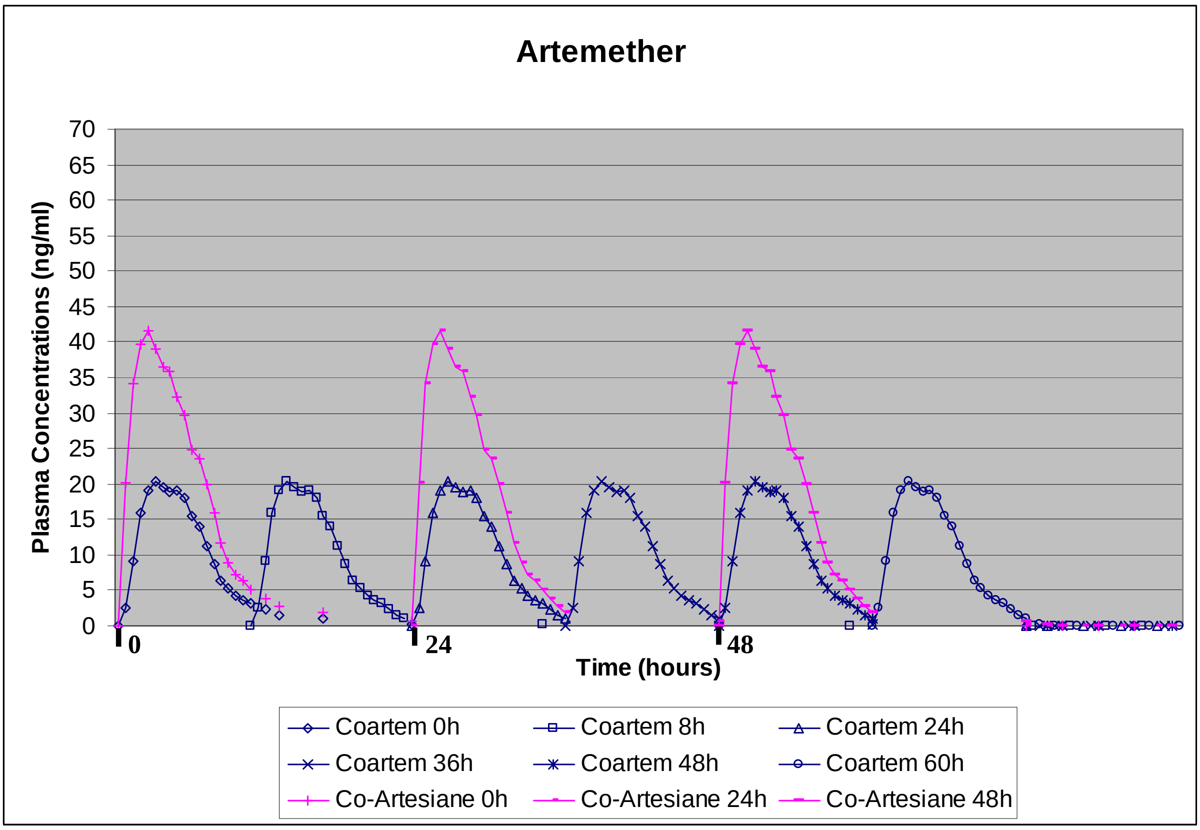

Artemether
Plasma Concentrations (ng/ml)
70
65
60
55
50
45
40
35
30
25
20
15
10
5
0
0
24
48
Time (hours)
Coartem 0h
Coartem 8h
Coartem 24h
Coartem 36h
Coartem 48h
Coartem 60h
Co-Artesiane 0h
Co-Artesiane 24h
Co-Artesiane 48h

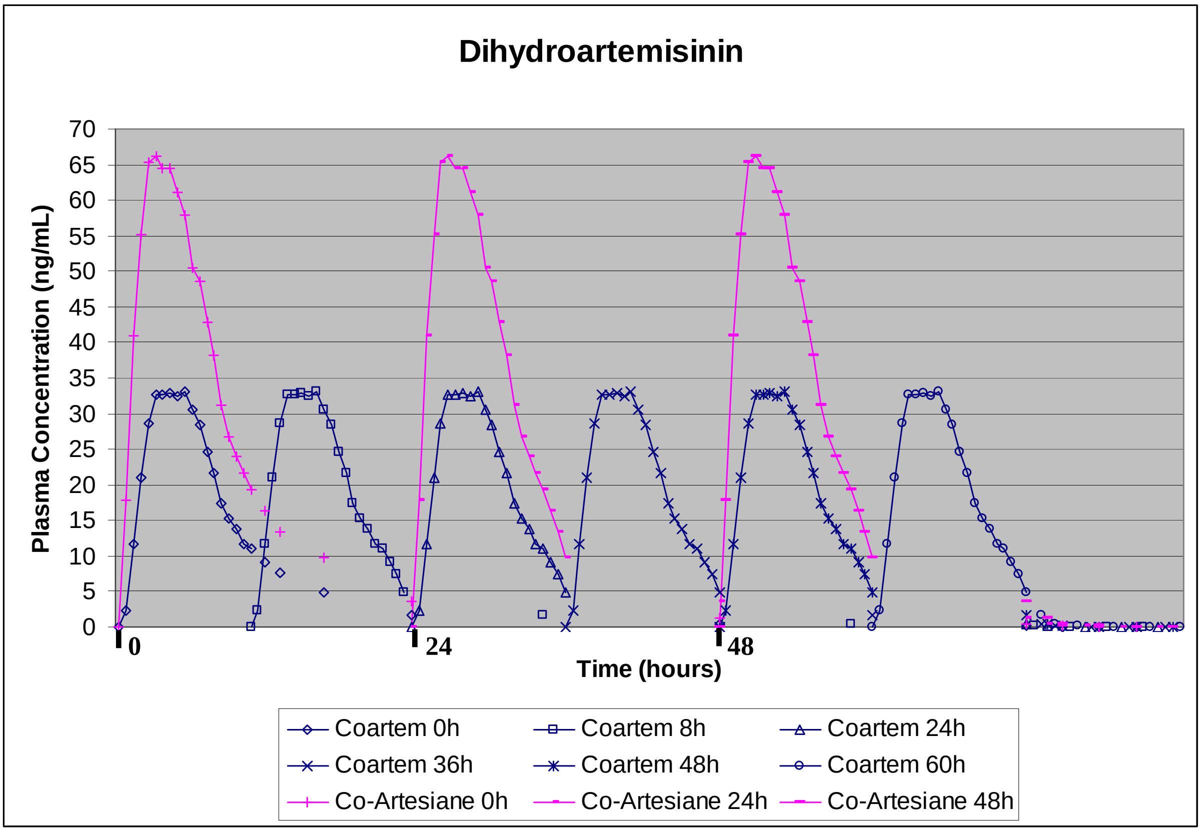

Dihydroartemisinin
Plasma Concentration (ng/mL)
70
65
60
55
50
45
40
35
30
25
20
15
10
5
0
0
24
48
Time (hours)
Coartem 0h
Coartem 8h
Coartem 24h
Coartem 36h
Coartem 48h
Coartem 60h
Co-Artesiane 0h
Co-Artesiane 24h
Co-Artesiane 48h

References

Ashley EA, Stepniewska K, Lindegardh N, McGready R, Annerberg A, Hutagalung R, Singtoroj T, Hla G, Brockman A, Proux S, Wilahphaingern J, Singhasivanon P, White NJ, Nosten F (2007) Pharmacokinetic study of artemether-lumefantrine given once daily for the treatment of uncomplicated multidrug-resistant falciparum malaria.Trop Med Int Health, 12(2):201-8.

Chanda P, Hawela M, Kango M, Sipilanyambe N (2006) Assessment of the therapeutic efficacy of a paediatric formulation of artemether-lumefantrine (Coartesiane) for the treatment of uncomplicated Plasmodium falciparum in children in Zambia. Malar J, 5:75

Newton P, Suputtamongkol Y, Teja-Isavadharm P, Pukrittayakamee S, Navaratnam V, Bates I, White N (2000) Antimalarial bioavailability and disposition of artesunate in acute falciparum malaria. Antimicrob Agents Chemother, 44(4):972-7

Silamut K, Newton PN, Teja-Isavadharm P, Suputtamongkol Y, Siriyanonda D, Rasameesoraj M, Pukrittayakamee S, White NJ (2003) Artemether bioavailability after oral or intramuscular administration in uncomplicated falciparum malaria.Antimicrob Agents Chemother, 47(12):3795-8)

World Health Organisation (2006) Guidelines for the treatment of malaria.
